# Ageing and endurance training effects on quantity and quality of pulmonary vascular bed in healthy men

**DOI:** 10.1186/1465-9921-15-8

**Published:** 2014-01-24

**Authors:** Ghanima Al Dandachi, Cécile Londner, Aurore Caumont-Prim, Laurent Plantier, Brigitte Chevalier-Bidaud, Jean-François Toussaint, François-Denis Desgorces, Christophe Delclaux

**Affiliations:** 1 AP-HP, Hôpital européen Georges-Pompidou, Service de Physiologie – Clinique de la Dyspnée, 75015 Paris, France; 2 AP-HP, Hôpital européen Georges-Pompidou, Unité d’Épidémiologie et de Recherche Clinique, 75015 Paris, France; 3 Institut de Recherche bioMédicale et d’Epidémiologie du Sport, INSEP, Paris, France; 4 Sorbonne Paris Cité, Faculté de médecine, Université Paris Descartes, 75006 Paris, France; 5 Université Paris Descartes, Sorbonne Paris Cité, EA2511, 75014 Paris, France; 6 CIC 9201 Plurithématique, Hôpital Européen Georges Pompidou, 75015 Paris, France; 7 Physiologie Respiratoire – Clinique de la Dyspnée, Hôpital européen Georges-Pompidou, 20, rue Leblanc, 75015 Paris, France

**Keywords:** Physical activity, Exercise, Capillary blood volume, Lung diffusion

## Abstract

It has recently been demonstrated that in healthy individuals, peak oxygen consumption is associated with a greater pulmonary capillary blood volume and a more distensible pulmonary circulation. Our cross-sectional study suggests that, in healthy men aged 20 to 60 years (n = 63), endurance sport practice (vigorous-intensity domain of the International Physical Activity Questionnaire) is associated with better quantity (pulmonary capillary blood volume) and quality (slope of increase in lung diffusion for carbon monoxide on exercise) of the pulmonary vascular bed, partly counterbalancing the deleterious effects of ageing, which remains to be demonstrated in a prospective longitudinal design.

## Findings

### Background

Pulmonary vascular response to exercise varies considerably from one individual to another [1], which is partly explained by the natural distensibility of the pulmonary circulation [2]. Capillary resistance significantly contributes to changes in pulmonary vascular resistance during exercise. Capillary blood volume available for gas exchange (VC) can be estimated from lung diffusing capacity measurements using both carbon monoxide (CO) and nitric oxide (NO) as tracer gases [3]. Lalande et al. recently demonstrated that in healthy individuals, peak oxygen consumption ($\dot{V}\;{O_{2}}_{,\mathrm{peak}}$) on exercise is associated with a greater pulmonary capillary blood volume and a more distensible pulmonary circulation [4], which is in keeping with the notion that a greater pulmonary vascular reserve allows for a higher aerobic exercise capacity, and vice-versa. On the opposite, ageing is associated with a progressive deterioration of the structure and function of the pulmonary vascular bed [5,6]. Using normative equations, one can calculate that VC would be zero around 100 years [7].

### Methods

**Figure 1:**
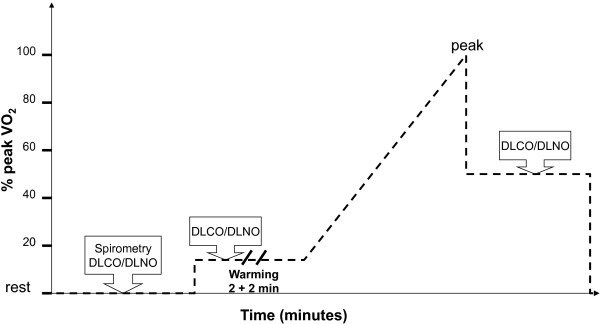
**Description of the investigations (experimental protocol).** Symptom-limited incremental exercise testing was conducted on an electronically braked cycle ergometer using the Vmax Cardiopulmonary Exercise Testing System (Sensor Medics, Yorba Linda, CA). After a 2 min warm-up period (inactive: 30 watts; trained subjects: 50 watts, corresponding to ~15% of $\dot{V}\;{O_{2}}_{,\mathrm{peak}}$), the workload was increased by 15–30 Watts/minute using a ramp protocol until exhaustion. The single breath DL_CO_/DL_NO_ measurements were obtained at rest and at two levels of exercise (a first warm-up period was performed before the ramp exercise test, and at 50% of the maximal work rate, immediately after peak exercise acquisition).

We thus enrolled both inactive and active (endurance-trained) healthy men to evaluate whether physical activity and age were correlated with VC at rest and with the increase in lung diffusion of CO (DL_CO_) on exercise. Physical activity was evaluated using the French version of the International Physical Activity Questionnaire (IPAQ, long form) [8] that evaluates vigorous, moderate and walking activity domains. The resting VC was calculated using DL_CO_ and lung diffusion of NO (DL_NO_) measured simultaneously by the single-breath technique (4 seconds breath-hold, corrected for haemoglobin, assuming negligible erythrocyte resistance to NO [9], using an automatic apparatus (MasterScreenBody, Jaeger), as previously described [10]). VC measurements were also obtained at two levels of exercise (warm-up period and at 50% of the maximal work rate: see Figure 1, experimental protocol) to calculate the slope of the DL_CO_/work relationship as an index evaluating the dynamic capillary blood volume recruitment/dilation on exercise. The linearity of the relationship between power and DL_CO_ has been demonstrated for ${\dot{V}}_{2}$ ≤60% of $\dot{V}\;{O_{2}}_{,\mathrm{peak}}$[11]. Symptom-limited incremental exercise testing was conducted on an electronically braked cycle ergometer according to the recommended guidelines [12], as previously described [13] using a 2 min warm-up period followed by a ramp protocol (see Figure 1 legend) until exhaustion. Spirometry was obtained before exercise.

### Results

A total of 64 healthy (no medication, never smokers or ex-smokers <5 pack-year, no history of asthma, between 40 and 60 years of age) Caucasian men were recruited: 32 inactive subjects (**not meeting specified American physical activity guidelines of at least** 1 hour and 15 minutes a week of vigorous-intensity aerobic physical activity, for five consecutive years) and 32 endurance-trained subjects (sport practice >3 hours/week for 5 consecutive years). One inactive man was unable to perform apneas for DL_CO_ measurements and was excluded. Informed written consent was obtained from all subjects, and ethical approval (CPP IDF VI, ID-RCB: 2011-A00006-35) was received.

**Figure 2:**
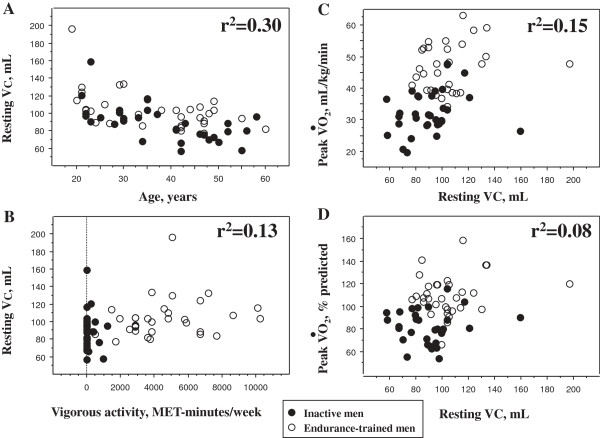
**Factors associated with resting capillary blood volume.** The panel **A** describes the relationship between age (years) and resting capillary blood volume available for gas exchange (VC, mL) in inactive (closed circles) and endurance-trained (open circles) men. A significant relationship is evidenced (see Table 2). The panel **B** describes the relationship between the vigorous-intensity activity domain of the International Physical Activity Questionnaire (Vigorous-IPAQ) and resting VC. A significant relationship is evidenced (see Table 2). The panel **C** describes the relationship between resting VC and $\dot{V}\;{O_{2}}_{,\mathrm{peak}}$ expressed as raw values, while the panel **D** describes the relationship between $\dot{V}\;{O_{2}}_{,\mathrm{peak}}$ normalized for age and height (expressed as% predicted, see Table fourteen in [12]). The statistical significance of the relationship of panel C is given in Table 2. The significance of the relationship of panel D (r = 0.311, p = 0.013) further suggests that VC is an independent (of age and height) predictor of $\dot{V}\;{O_{2}}_{,\mathrm{peak}}$.

The main characteristics of the healthy men at rest and on exercise are described in Table 1 while univariate analyses of factors associated with the quantity and quality indices of pulmonary vascular bed are described in Table 2 and Figure 2. Multivariate analyses (age, height and activity as independent variables) demonstrated that both age and vigorous-intensity activity domain remained independent predictors of resting VC (R^2^ = 0.37, p-value (vigorous intensity activity) = 0.0123, p-value (age) <0.001), and of the slope of DL_CO_ increase (R^2^ = 0.25).

**Table 1:** Characteristics of the healthy men

**Characteristics n median [interquartile]**	**Healthy men n = 63**	**Inactive men n = 31**	**Trained men n = 32**	**P value #**
Age, years	38 [27; 47]	37 [29; 48]	39 [25; 46]	0.574
Height, cm	179 [173; 185]	177 [171; 183]	180 [178; 186]	0.038
Haemoglobin, g/dL	14.4 [13.7; 15.0]	14.4 [13.7; 15.4]	14.3 [13.7; 14.5]	0.397
**Physical activity assessment**				
Sport practice, hours/week	4.0 [0.0; 8.0]	0.0 [0.0; 0.0]	7.0 [5.0; 10.0]	<0.001
Vigorous-intensity domain, MET-min/week	1200 [0; 3840]	0 [0; 80]	3840 [2880; 6300]	<0.001
Total IPAQ score, MET-min/week	3577 [1105; 7482]	1099 [684; 2739]	6943 [3909; 9887]	<0.001
**Resting values**				
FEV_1_,% predicted	111 [103; 119]	108 [99; 116]	113 [105; 122]	0.137
DL_CO_, mmol/min/kPa	10.84 [9.52; 11.68]	10.51 [8.55; 11.19]	11.29 [10.21; 12.08]	0.005
VC, mL	95 [82; 104]	89 [76; 99]	103 [89; 112]	0.002
DM_CO_, mmol/min/kPa	22.53 [21.51; 23.64]	21.31 [19.80; 22.81]	23.69 [21.18; 25.67]	0.019
**At 50% peak work load**				
DL_CO_, mmol/min/kPa	13.42 [12.81; 14.05]	11.90 [10.67; 13.45]	14.23 [13.08; 16.08]	0.001
VC, mL	125 [117; 132]	108 [95; 126]	129 [110; 153]	0.025
DM_CO_, mmol/min/kPa	25.42 [24.31; 26.44]	23.41 [21.90; 24.92]	27.33 [25.81; 28.84]	< 0.001
**At peak exercise**				
Exercise duration, min	640 [589; 721]	640 [592; 735]	632 [585; 688]	0.640
$\dot{V}\;{O_{2}}_{,}$ mL/kg/min	2932 [2370; 3600]	2385 [2068; 2671]	3468 [3181; 3900]	<0.001
$\dot{V}\;{O_{2}}_{,}$% predicted	95 [80; 108]	80 [68; 93]	107 [97; 119]	< 0.001

#using Mann and Whitney U test.

VC denotes capillary blood volume and DM_CO_ denotes the pulmonary membrane diffusing capacity for carbon monoxide calculated using single breath DL_CO_/DL_NO_ measurement.

**Table 2:** **Univariate analyses assessing the factors associated with resting capillary blood volume and slope of DL**_**CO**_**/work relationship**

	**Resting capillary blood volume, mL**		**Slope of DL**_**CO**_**/work relationship, mmol/min/kPa/% peak work rate**	
**Linear regression, pearson**	**r value**	**P value**	**r value**	**P value**
Age, years	-0.545	<0.001	-0.258	0.041
Height, cm	0.537	<0.001	0.324	0.009
Peak $\dot{V}\;{O_{2}}_{,}$ mL/kg/min	0.390#	0.002	0.244	0.045
Sport practice, hours/week*	0.360	0.006	0.278	0.034
Vigorous-IPAQ, MET-min/week	0.356	0.004	0.264	0.036
Total-IPAQ, MET-min/week	0.116	0.364	0.218	0.086

*sports were rowing (=19) triathlon (n = 6) and miscellaneous (n = 7) for the 32 endurance-trained men. The Vigorous-IPAQ domain was clearly related to sport practice duration (r^2^ = 0.64; p < 0.0001).

#: the Pearson coefficient increased to 0.53 after excluding the two outliers using the Tietjen and Moore method (that is consistent with the r value of 0.60 for the same relationship in the study of Lalande et al. [4]).

### Discussion

Lalande et al. recently demonstrated that $\dot{V}\;{O_{2}}_{,\mathrm{peak}}$ is associated with a greater VC and with a more distensible pulmonary circulation [4] that deserved to be confirmed. We enrolled a larger sample of healthy subjects with a wide range of physical activity levels allowing to establish the relationship between physical activity and $\dot{V}\;{O_{2}}_{,\mathrm{peak}}$ and to perform a multivariate analysis more confidently. Different methods for VC measurement have been used with and without inhaled NO; their agreement is satisfactory [14]. The degree of both DL_CO_ and VC increase during exercise (~20-25%) in our study was similar with that described by Lalande et al. [4]. Overall, the level of statistical significance of the correlations evidenced is weak to moderate. Several factors may explain this finding such as genetic heritability and inherent limitations due to indirect measures of vascular indices (with two outliers, see Figure 1). The genetic heritability of $\dot{V}\;{O_{2}}_{,\mathrm{peak}}$ is around 50% [15], which may explain the overlap between $\dot{V}\;{O_{2}}_{,\mathrm{peak}}$ is evidenced in inactive and trained men in our study, and further justify the weak relationship between physical activity and vascular indices.

The results of this cross-sectional study rely on correlations that do not make causality. Nevertheless, one may hypothesize that endurance sport practice favours lung growth and capillary blood volume increase as observed for lung volumes [16,17].

In conclusion, our cross-sectional study suggests that, in healthy men aged 20 to 60 years, endurance sport practice is associated with better quantity and quality of the pulmonary vascular bed, partly counterbalancing the deleterious effects of ageing, which remains to be demonstrated in a prospective longitudinal design.

## Abbreviations

VC: capillary blood volume available for gas exchange; CO: carbon monoxide; NO: nitric oxide; $\dot{V}\;O_{2}$: _peak,_ peak oxygen consumption; DLCO: lung diffusion of CO; IPAQm: International Physical Activity Questionnaire; DLNO: lung diffusion of NO

## Competing interests

All the authors declare that they have no competing interests.

## Authors’ contribution

All the authors made substantial contributions to conception and design (JFT, FDD, CD2), or acquisition of data (GAD, CL, LP, BCB), or analysis and interpretation of data (ACP); drafted the submitted article or revised it critically for important intellectual content (all authors); and provided final approval of the version to be published (all authors).
